# The Effect of Cooling Fluid Composition on Ablation Size in Hepatic Laser Ablation: A Comparative Study in an Ex Vivo Bovine Setting

**DOI:** 10.3390/tomography9050131

**Published:** 2023-09-01

**Authors:** Fiona Mankertz, Nadine Bayerl, Ole Gemeinhardt, Norbert Hosten, Marie-Luise Kromrey

**Affiliations:** 1 Institute for Diagnostic Radiology and Neuroradiology, University Medicine Greifswald, 17475 Greifswald, Germany; 2 Institute of Radiology, University Hospital Erlangen, Friedrich-Alexander-Universität (FAU) Erlangen-Nürnberg, 91054 Erlangen, Germany; 3 Department of Radiology, Charité—Universitätsmedizin Berlin, 10117 Berlin, Germany

**Keywords:** laser ablation, hyperthermic ablation, interventional radiology, experimental radiology, oncoradiology

## Abstract

Purpose: Hyperthermic ablation is a minimally invasive mode of tumour therapy which serves as a viable alternative to surgical intervention. However, one of the major drawbacks, besides the heat sink effect and the risk of damaging adjacent organs, is limited ablation size. The use of a cooling fluid during ablation has been shown to increase the ablation volume and decrease the carbonisation rate. The aim of this study was to investigate whether the composition of the cooling fluid has an effect on ablation size and carbonisation rate during hepatic laser ablation in an ex vivo bovine setting. Method: In this study bovine hepatic tissue was ablated in an ex vivo setting using an internally cooled laser applicator. A total of 45 tissue samples were assigned to three groups: 0.9% saline infusion (n = 15), distilled water infusion (n = 15) and a 50%/50% mixture of 0.9% saline and distilled water (n = 15). Ablation was conducted using a 1064 nm Nd:YAG laser at a wattage of 25 W and time interval of 10 min. The ablation volume and carbonisation rate were then measured and recorded through postprocedural MRI. One-way ANOVA and post-hoc testing were performed to assess the effect of the cooling fluid composition on the ablation volumes. Results: We found that using a mixture of saline and distilled water as a cooling fluid during hyperthermic ablation resulted in a larger ablation volume (mean ± SD: 22.64 ± 0.99 cm^3^) when compared to saline infusion (21.08 ± 1.11 cm^3^) or distilled water infusion (20.92 ± 0.92 cm^3^). This difference was highly significant (*p* < 0.001). There was no significant difference in ablation size between the saline group and the distilled water group. The highest carbonisation rate occurred in the saline group (12/15), followed by the mixed infusion group (3/15) and the distilled water group (1/15). Conclusions: The results of this study suggest that cooling fluid composition during hepatic laser ablation affects ablation volume in an ex vivo bovine setting. There was no statistically significant difference when comparing ablation volumes during saline infusion and distilled water infusion, but the carbonisation rate was significantly higher when using saline. The combination of saline and distilled water in a 50%/50% mixture as cooling fluid appears to be an auspicious alternative, as ablation volumes created with it are larger when compared to saline and distilled water alone, while carbonisation rate remains low. This might improve patient outcome as well as patient eligibility for hyperthermic ablation.

## 1. Introduction

Thermal ablation is well established as the treatment of choice in very early and early-stage hepatocellular carcinoma (HCC) in Europe. According to the Barcelona Clinic Liver Cancer’s staging system, hyperthermic treatment through radiofrequency ablation (RFA) and microwave ablation (MWA) can be considered equivalent to surgical hepatectomy in certain cases [1]. However, this is limited to less than three tumour nodules each measuring ≤ 3 cm. According to the 2022 German S3 Guidelines, primary treatment through hyperthermic ablation is only strongly recommended for patients with HCC < 3 cm and compromised liver function [2]. In the US, RFA and MWA are considered an alternative treatment only if patients expressly decline surgical resection or are considered ineligible. However, hyperthermic ablation is still given a strong recommendation in the American Association for the Study of Liver Diseases guidelines (Level 1, Strong Recommendation) [3]. Similarly, treatment of liver metastases from colorectal cancer (CRC) by means of hyperthermic ablation is considered a viable alternative to surgical resection in certain settings such as non-resectable liver metastases, reduced general health of the patient, preceding resection of liver metastasis or in combination with resection [2,4,5,6,7].

The main factor limiting eligibility for ablation therapy is the maximum attainable ablation volume. This determines which tumours classify for hyperthermic ablation [8,9,10]. HCC tumours of 3 cm or less are considered curatively treatable through a single-applicator session of hyperthermic ablation such as RFA, MWA or laser ablation (LA) [11]. Larger HCC tumours require multiple sessions, multiple applicators or combination with other treatment modalities. However, in non-hepatic organ tissue, current modalities of hyperthermic ablation are unable to achieve ablation sizes larger than 3 cm (lung, RFA) or 2 cm (pancreas, MWA) [3,4]. The extent of ablation is limited by the conversion of viable energy-conducting tissue to a less efficient energy-conducting insulating boundary layer, which is caused by carbonisation during hyperthermic ablation. Once tissue temperatures surpass 100 °C, charring tissue creates an insulating rim that impedes outward heat spread and limits ablation size [12,13,14]. A promising approach to increase ablation size is to prevent or minimize carbonisation of the treated tissue [15]. Therefore, identifying methods to enhance ablation volume, for example, by limiting carbonisation, has the potential to improve patient outcomes.

Another phenomenon that limits ablation size in hyperthermic ablation is the heat sink effect. This effect is especially poignant in lesions near larger vascular structures where continuous circulation causes a cooling effect [16] and reduces tissue temperatures. As such ablation volume is limited, several methods to combat the limiting factor of the heat sink effect during hyperthermic ablation have been developed. MWA has been reported to be less susceptible to the heat sink effect than RFA, and its ablation volume is therefore larger [17]. Another approach addresses the heat sink effect by reducing arterial blood flow, which is a complex and risky but promising procedure [12]. Occlusion of portal venous blood flow by using the Pringle manoeuvre, intraarterial embolisation or balloon occlusion can increase ablation size. However, it carries serious risks regarding ischaemia of the liver itself, damage to other organs, intraoperative haemorrhage or post-interventional ischaemia reperfusion syndrome [18,19,20]. A less invasive and therefore less risky, well-established method is the infusion of cooling fluid through an internally cooled applicator. This has been successfully demonstrated in microwave, radiofrequency and laser ablation [21,22,23]. Ablation volume in RFA increased by approximately 2 cm when an internally cooled applicator was utilised in combination with a high-power interval pulse [24,25]. However, the effect of cooling fluid composition on ablation volume and carbonisation rate has not been extensively studied. In a 1998 study by Vogl et al., the use of a standardised 0.9% saline solution for laser ablation was noted. However, the authors did not elaborate further on the choice of cooling fluid [21]. While several studies using RFA have compared the efficacy of 0.9% saline infusion and 0.45% half-normal saline infusion in cardiac ablation, their long-term results have not been significant [26]. Only two older studies mention utilizing fluids other than standardised 0.9% saline: A study conducted by Ishikawa et al. in 2013 found that 0.9% saline infusion increased ablation size when compared to 50% glucose infusion [27]. This is contrasted by a 2008 study which found a significant increase in ablation size when using 5% glucose infusion, with the smallest ablation size observed when using 0.9% saline infusion [28]. There is, in short, a lack of data regarding the effect of cooling fluid composition on ablation volume and carbonisation rate in hyperthermic ablation.

As shown in a recent proof-of-concept study, a further method of increasing ablation volume in laser ablation through the prevention or delay of carbonisation is the use of a spacer, which has been linked to significantly delayed carbonisation and thus significantly increased ablation size [29]. Composition of the cooling fluid and its effect on ablation volume could thus be used to further optimize hyperthermic ablation. By using the most appropriate cooling fluid the problem of the limited ablation size might be more effectively addressed in future studies.

The purpose of this study was to improve upon the lack of data on cooling fluid composition and its effect on ablation size and carbonisation rate. In clinical practice, 0.9% saline solution is used as a standardised infusion fluid due to its low cost, availability and lack of absolute contraindications. However, the lack of scientific data impedes any evaluation of its efficacy. Therefore, we investigated the effect of cooling fluid composition on the ablation volume during hepatic hyperthermic ablation in an ex vivo bovine setting.

## 2. Materials and Methods

A total of forty-five ablation procedures were conducted on bovine hepatic tissue in an ex vivo setting. Whole bovine livers were obtained from a food-grade abattoir (LandWertHof Stahlbrode, Sundhagen, Germany) within one hour after slaughtering. No animals were specifically slaughtered for the purpose of this trial, and as such, according to the ARRIVE guidelines for animal research, no consultation of the local ethics committee was required [30]. The livers were transported in a heat-resistant polystyrene box at room temperature. A certified veterinarian conducted a prior inspection to achieve uniformity of tissue by removing any appendages such as omental fat. Large blood and bile conducting structures (e.g., portal vein, vena cava, hepatic artery, and bile ducts) were longitudinally sectioned to impede the diffusion of fluids into the air-filled vessels. The prepared livers were then used for the ablation procedures. Exclusion criteria for ablation of hepatic tissue were <200 mm tissue depth, <100 mm margin to main hepatic veins or lobar portal veins, <100 mm margin to the capsule and caudate lobus. The ablation procedure was conducted on the livers as a whole.

We chose a hyperthermic energy-based mode of ablation: laser ablation. The biomechanics of laser ablation have been abundantly discussed in numerous studies [31,32]. Laser ablation allows a clear macroscopical and imaging-based differentiation between vital liver tissue and ablated, avital liver tissue, which has been confirmed in previous research [33]. For the ablation procedure itself, a Medilas Fibertom 5100 (Dornier Medtech Europe GmbH, Munich, Germany) was combined with a standard RoweCath applicator (RoweMed, Parchim, Germany), whose utilization has been saliently described in previous academic literature [23]. The applicator was positioned blindly in the ex vivo tissue of the bovine livers as no specific lesion needed to be treated. Precautions included choosing a sufficient tissue border on each side to prevent ablation zones reaching the liver capsule. The ablation zone and carbonisation were to be demonstrated on postprocedural MRI.

The laser applicator was internally cooled through the infusion of different types of cooling fluids. Our independent variable “fluid type” was subdivided into three groups: We first conducted the ablation procedure using 100% isotonic saline fluid (9 g sodium chloride per 1 L water) at a flow rate of 60 mL/h (n = 15). Our second group of ablation procedures was conducted using 100% distilled water at a flow rate of 60 mL/h (n = 15). Finally, to measure the influence of each group, we conducted a third set of ablation procedures using a mixture of 50% isotonic saline fluid and 50% distilled water at a flow rate of 60 mL/h (n = 15).

Ablation volume was defined as the dependent variable. Our set of volumetric data was obtained through MR-based volumetry after laser ablation. Postprocedural MR-based monitoring with its clear imaging, thermometric capabilities and sequence-based motion correction [34,35], along with CT and ultrasound, is one of the most effective tools for assessing ablation success within the first 24 h after ablation procedures [36]. As discussed in a preceding study, the comparison of manual volumetry through displacement of water and MR-based volumetry shows no statistically significant difference [29]. As such, MR-based volumetry is well suited as a non-invasive measuring method.

Post-ablation MRI was performed using a 7T BioSpec 70/30 (Bruker Corp., Billerica, MA, USA), Bruker BioSpin MRI slider (Bio Mouse Bed T2 Slider, model number T10211) and ¹H receive-only 8 × 1 mouse body surface array coil. The ablation zones were excised from the whole liver including a minimum diameter of 50 mm healthy, non-ablated tissue on each side of the ablation.

The ablated liver parcel was then wrapped in cellophane and carefully compressed. This prevented false positive inflation of the ablation volume due to cooling fluid accumulating in the ex vivo liver tissue lacking venous drainage. The liver was then placed on the MRI slider.

We acquired three MRI sequences in the axial and sagittal planes suitable for evaluation of tissue after ablation according to Ozkavukcu et al. [37]: T1-weighted FL3D VIBE (axial and sagittal planes TE/TR 1.5/8.0 milliseconds each), T1-weighted FL2D (axial plane: TE/TR 4.3/365 milliseconds, sagittal plane: TE/TR 4.3/274.0 milliseconds) and T2-weighted TSE (axial plane: TE/TR 9.2/160 milliseconds, sagittal plane: TE/TR 32.0/1410.0 milliseconds). The inclusion criteria for volumetry after the successfully completed ablation was visible T1-weighted hyperintensity of the ablation zone as opposed to the non-ablated tissue. The inclusion criteria were confirmed by two independent readers. Axial T1-weighted FL2D sequences were found to be the most suitable sequences for identification and measurement of the ablation zone and were therefore used for subsequent volumetry.

Manual volumetry of each liver was performed by using ROI measurements on the axial T1-weighted FL2D sequences showing the hyperintensity of the ablation zone by contouring the hyperintense area point by point using the “closed polygon” tool of Horos DICOM 4.0.0. Thus, the acquired two-dimensional area in square centimetres (cm^2^) was multiplied by the slice thickness to obtain the ablation volume in cubic centimetres (cm^3^). Finally, all calculated ablation volumes per slice were summed up to obtain the total ablation zone volume. Additionally, a second semi-automatic method of volumetry was extended through the Horos DICOM 4.0.0 viewer’s “Add Missing ROIs” AI feature, where the reader outlined a ROI on an exemplary slice and the missing ROIs can then be automatically generated. This feature allowed a semi-automatic calculation of the ablation volume and reduced time required for evaluation as opposed to fully manual volumetry.

The rate of carbonisation was assessed on a binomial scale (yes/no), whether carbonisation was visible or not. Carbonisation is visualised as T1-weighted hypointense area at the previous applicator site within the T1-weighted hyperintense ablation zone. Again, as previously described for the identification of the hyperintense ablation zone, the axial T1-weighted FL2D sequences were used to assess the presence of carbonisation, as this sequence produced the best visibility.

Statistical analysis was performed using SPSS (SPSS Statistics for Windows, version 26.0, IBM Corp., Armonk, NY, USA). We chose one-way ANOVA as the most suitable testing method according to the UCLA Statistical Consulting Group [38]. One-way ANOVA is the testing method of choice when selecting one independent variable with two or more levels and dependent variables of a metric nature. As the requirement of successful calculation of one-way ANOVAs is the assumption of normal distribution of the dependent variable’s data sets, this assumption was first evaluated through Shapiro–Wilks and Kolmogorov–Smirnov when a significance level of *p* < 0.05 was set [39]. We additionally calculated the intra-class correlation coefficient (ICC) to assess intra-rater reliability.

For the independent variable “fluid type”, the three levels “saline”, “distilled water” and “saline-water mixture” with 15 entries each (n = 45 total) were tested for the presence of statistical significance. Post hoc testing was then performed to gauge the quality of statistical significance. We adhered to Field et al.’s recommendation of using the Tukey post hoc in data sets where group variances and sample sizes are closely similar [40]. For post hoc testing, a significance level of *p* < 0.05 was assumed.

## 3. Results

We found that using a mixture of 0.9% saline and distilled water as a cooling fluid during hepatic laser ablation resulted in a mean ablation volume of 22.64 cm^3^ with a standard deviation of ±0.99 cm^3^. The mean ablation volume of the 0.9% saline infusion group was 21.08 ± 1.11 cm^3^. The distilled water infusion group had a mean ablation volume of 20.92 ± 0.92 cm^3^ (Table 1 and Table 2).

The group in which 0.9% saline was infused during ablation showed the highest carbonisation rate with 12/15 tissue samples showing signs of carbonisation (80%). The group in which a mixture of 50/50 saline and distilled water in contrast included 3/15 tissue samples with carbonisation (20%). Lastly, the distilled water group included one tissue sample showing signs of carbonisation out of a total of n = 15 (6.67%).

When examining the three infusion groups regarding the dependent variable of ablation volume through one-way ANOVA in a test of between-subjects effects, we found that statistical significance was present at a corrected model and intercept of *p* < 0.001. We then conducted Tukey post-hoc testing to determine the quality of that statistical significance and to investigate between which groups statistical significance was present.

In post-hoc testing we observed no statistically significant difference between the distilled water infusion group and the 0.9% saline group at a significance level of *p* < 0.05. At a significance level of 0.908, both groups showed a very similar distribution of ablation volume. In contrast, the third group, in which the 50/50 mixture of saline and distilled water was used as a cooling fluid during ablation, showed a highly significant statistical difference of *p* < 0.001, when compared to the 0.9% saline group. There was also a statistically significant difference when comparing the mixture of saline and distilled water group to the distilled water group (*p* < 0.001). This mean difference of the mixture group was a net positive of +1.56 cm^3^ when compared to the 0.9% saline group and +1.72 cm^3^ when compared to the distilled water group (Table 2). The intraclass correlation coefficient was calculated to be 0.848 through SPSS, indicating good intra-rater reliability.

We additionally investigated the carbonisation rate. Statistical significance was present here as well at a corrected model and intercept of *p* < 0.001. The carbonisation rate in the group using a 50/50 mixture of saline and distilled water as cooling fluid was significantly lower than using 0.9% saline (*p* < 0.001), and there was no statistical difference compared to the distilled water group (*p* = 0.588). We also observed that the carbonisation rate in the group using distilled water as a cooling fluid was significantly lower compared to using 0.9% saline (*p* < 0.001, Table 2).

**Figure 1:**
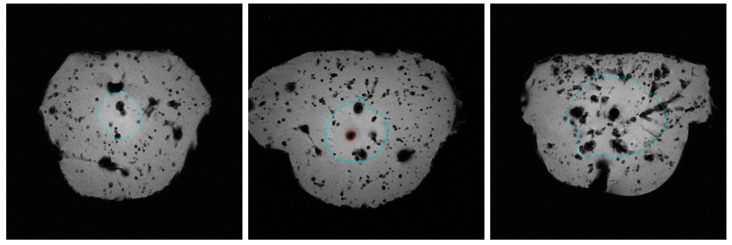
T1-weighted (FL2D) MRI visualising ablation margin (turquoise) and carbonisation margin (red) in three different infusion groups of cooling fluids: distilled water (**left**), 0.9% saline (**middle**) and saline-water mixture (**right**).

Figure 1 demonstrates the ablation margins (turquoise) and carbonisation margins (red) in the T1-weighted MRI of the liver. A higher-quality resolution to showcase carbonisation is available below. All three liver tissue samples feature homogenous fluid diffusion throughout the tissue, which confirms a successful infusion of the cooling fluid. Ablation margins are highlighted in turquoise. All three ablation margins are smooth and in a near-spherical shape. The left figure depicts a non-carbonised ablation margin when distilled water was infused. It is comparatively smaller than the other two ablation volumes. The middle figure has a comparatively larger ablation margin but also shows a margin of carbonisation, which is highlighted in red. This is a sample where 0.9% saline was infused during ablation. The right figure illustrates a tissue sample where the 50/50 mixture of 0.9% saline and distilled water was infused during ablation. Its ablation margin is again comparatively larger, and there is no carbonisation.

## 4. Discussion

The purpose of this study was to investigate the effect of cooling fluid composition such as 0.9% saline and distilled water on the ablation volume and carbonisation rate during hepatic laser ablation in an ex vivo bovine setting using postprocedural MR-based monitoring. Up to this point, there have been very few studies evaluating the relationship between the choice of cooling fluid during hyperthermic ablation and ablation size. However, there is ample scientific evidence certifying the fact that the presence of cooling fluid significantly increases ablation size [27,28,41]. It therefore stands to reason that modifying the composition of cooling fluid might also modify its outcome. In synopsis, we found that this was indeed the case: the composition of the cooling fluid during hepatic laser ablation in an ex vivo bovine model was able to significantly affect ablation volume based on postprocedural MR-monitoring.

In clinical practice, 0.9% saline is a commonly used infusion fluid during hyperthermic ablation, as reported in various studies. Hyperthermic ablation dates back to the early 1990s, during which saline was already used. In a then-novel 1999 study, Francica and Marone reported the use of saline during radiofrequency ablation [42]. Huang et al. further emphasized the use of saline as a standard practice in a 2022 study and highlighted its insulating properties for protection of adjacent tissue [43]. Kho et al., in a 2021 experimental study, evaluated the effect of saline concentration during radiofrequency ablation on electrical conductivity and carbonisation rate, finding that a saline concentration of 15% produced the highest electrical conductivity. At the same time, this 15% saline concentration also led to an increased rate of carbonisation [44].

**Figure 2:**
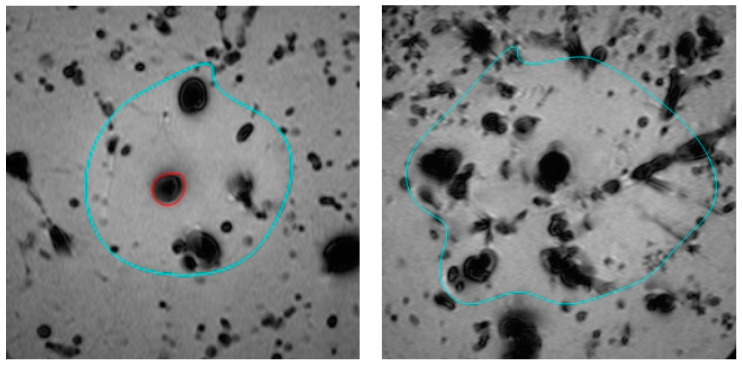
Tissue carbonisation (red) and ablation margin (turquoise) during laser ablation visualised in postprocedural T1-weighted MRI of the liver: 0.9% saline cooling fluid (**left**) and a mixture of 0.9% saline and distilled water (**right**).

In this study, we compared the effect of using 0.9% saline versus distilled water as the cooling infusion fluid during hepatic laser ablation using postprocedural MR-monitoring. Although there was no significant difference in ablation volume between the two groups, the use of 0.9% saline resulted in a significantly higher rate of carbonisation, with 12 out of 15 cases showing carbonisation in the 0.9% saline infusion group, compared to only 1 out of 15 cases in the distilled water group (*p* < 0.001). Figure 2 displays the slim border of carbonisation (highlighted in red on the left) when using 0.9% saline as an infusion fluid, whereas there is no carbonisation when using a mixture of 0.9% saline and distilled water (on the right). Our findings suggest that while 0.9% saline may be a trusted choice for infusion fluid, its higher rate of carbonisation limits ablation volume.

The use of other cooling fluids in hyperthermic ablation has heretofore not been widely explored in scientific literature. Laeske et al. discuss the advantages of 5% glucose infusion during radiofrequency ablation in the context of preventing diaphragmatic or pulmonal injury in clinical practice but do not evaluate its impact on ablation volume [45]. To the authors’ knowledge, the use of distilled water or a mixture of 0.9% saline and distilled water is a novel method in hyperthermic ablation.

Our findings support the initial hypothesis that the composition of cooling fluid used has a significant impact on ablation volume. Whereas there was no statistically significant difference between the 0.9% saline infusion group and the distilled water infusion group, the group in which a 50/50 mixture of 0.9% saline and distilled water was used showed a difference in ablation volume at a significance level of *p* < 0.001. This level of significance was present both when comparing the 50/50 mixture group to the 0.9% saline group as well as comparing the 50/50 mixture group to the distilled water group. When drawing on this difference, it can be concluded that the sole use of 0.9% saline in a clinical setting limits ablation size and could be improved upon. Additionally, the rate of carbonisation when using a 50/50 mixture of 0.9% saline and distilled water also resulted in a significantly lower rate of carbonisation, impacting only 3/15 tissue samples (*p* < 0.001), compared to the 12/15 positive rate of carbonisation in the 0.9% saline infusion group. This results in an absolute risk reduction of causing carbonisation of 60%, or a relative risk reduction of 75%. It is known that the thermal conductivity of a fluid depends on its ion concentration, providing a possible explanation for the dependence of ablation size and carbonisation on the composition of the cooling fluid [44]. However, as the optimum level for achieving larger ablation volumes in our study was determined to be a salt concentration of 0.45% using a 50/50 mixture of 0.9% saline and distilled water, there appears to be at least one other yet unidentified influencing component in addition to the effects mediated by thermal conductivity.

The relation between carbonisation and ablation size has been extensively discussed in an author’s previous study [29]. It can be concluded that minimising the chance of carbonisation leads to an increase in ablation rate, which is beneficial in a clinical setting. For larger-sized tumours, conventional surgical resection is generally considered preferable to ablation therapy. Tumour ablation is limited to smaller, solitary nodules. This is due to the risk of incomplete ablation and thus the chance of malignant tissue remaining. This problem can be countered by using multiple applicators. However, with every additional applicator the risk of tissue perforation, vascular haemorrhaging as well as inhomogeneity of the ablation zone increases. Pruitt et al. noted an increased complication rate when using more than a single applicator and recommended limiting the maximum number of applicators during hyperthermic ablation [28].

Our study’s limitations are its small sample size, the ex vivo setting and its use of bovine liver rather than human tissue. In addition, the circulatory heat sink effect is missing in an ex vivo study. Therefore, we plan on conducting further testing on in vivo porcine hepatic tissue in a future study. An alternative might be the use of an artificial perfusion machine similar to that developed by Koch et al., which simulates hepatic perfusion during hyperthermic ablation [46]. This would allow the accurate assessment of the influence of fluid composition in a pseudo-in vivo setting using human tissue.

**Figure 3:**
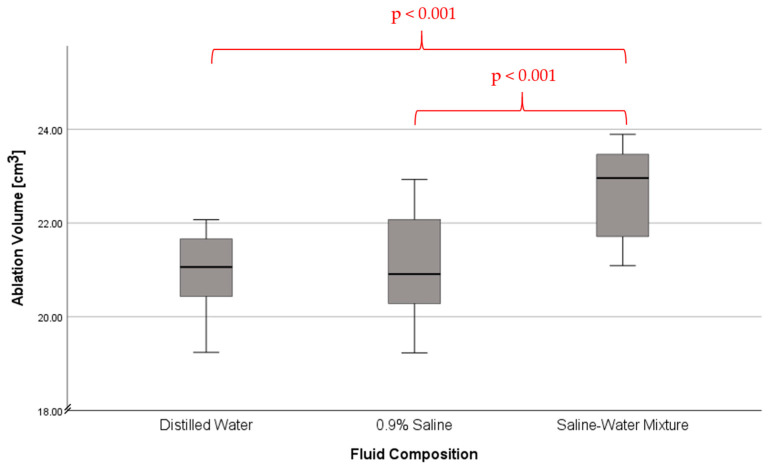
Ablation volume in laser ablation depending on fluid composition. Whiskers indicating error bar width.

Additionally, while statistical significance is present, the difference is comparatively small (Figure 3). As such, we cannot conclude that a mixture of 0.9% saline and distilled water is optimal for hyperthermic ablation, merely that it is slightly better than the current status quo. Despite this, the presence of statistical significance confers that cooling fluid composition has a relevant effect on ablation volume.

We investigated only three different compositions of cooling fluids (0.9% saline, distilled water, and a 50/50 mixture of 0.9% saline and distilled water). Therefore, we can only demonstrate which of the three compositions used attains best results in terms of maximum ablation size and minimum carbonisation rate. In our study, this was concluded to be the 50/50 mixture of 0.9% saline and distilled water as a cooling fluid. There may be other more appropriate fluid compositions that we have not yet investigated and may show a larger margin of difference. Future studies may examine fluids such as hypertonic saline, glucose solution or copper sulfide.

## 5. Conclusions

We demonstrated that cooling fluid composition affects ablation size and carbonisation rate during hepatic laser ablation in an ex vivo bovine setting as monitored by postprocedural MRI volumetry. We found that a 50/50 mixture of 0.9% saline and distilled water as a cooling fluid significantly increases ablation volume while minimising the carbonisation rate when compared to 0.9% saline and distilled water alone. No significant differences in ablation volumes were observed between saline infusion and distilled water infusion as cooling fluids. Using saline as a cooling fluid resulted in higher carbonisation rates. As carbonisation acts as an insulating boundary that limits heat diffusion and therefore ablation size, any method that prevents or minimises its formation may allow larger ablation volumes and improve patient outcomes. Knowing that the composition of the cooling fluid can have a positive effect on ablation size, further research can be conducted to determine the most appropriate cooling fluid composition to achieve minimal carbonisation and larger ablation volumes. This could improve patient outcomes, as the use of multiple applicators during ablation or multiple applications to treat larger organ lesions has been associated with a significant increase in peri- and postprocedural complications. The ability to create larger ablation volumes may also increase patient eligibility for hyperthermic ablation. The promising results that fluid composition can positively affect ablation volume in laser ablation require further development and validation in a clinical setting.

## Figures and Tables

**Table 1:** Descriptive statistics comparing ablation volume [cm^3^] in relation to fluid composition.

Fluid Composition		Ablation Volume [cm^3^]	Std. Error
Distilled Water	95% CI Lower Bound	20.41	0.24
	95% CI Upper Bound	21.43	
	Median	21.06	
	Minimum	19.24	
	Maximum	22.07	
	Mean	20.92	
	Std. Deviation	0.92	
0.9% Saline	95% CI Lower Bound	20.47	0.29
	95% CI Upper Bound	21.69	
	Median	20.91	
	Minimum	19.23	
	Maximum	22.93	
	Mean	21.08	
	Std. Deviation	1.11	
Saline-Water Mixture	95% CI Lower Bound	22.09	0.26
	95% CI Upper Bound	23.19	
	Median	22.96	
	Minimum	21.09	
	Maximum	23.89	
	Mean	22.64	
	Std. Deviation	0.99	

**Table 2:** Statistical analysis of mean ablation volume (cm^3^) and carbonisation (binary 0/1) in relation to fluid composition.

(I_A_) Fluid Composition	(J_A_) Fluid Composition	Mean Difference (I_A_ − J_A_)	Std. Error	Sig.	95% Confidence Interval	
					Lower Bound	Upper Bound
Distilled Water	0.9% Saline	−0.15	0.37	0.908	−1.05	0.74
	Saline-Water Mixture	−1.71	0.37	<0.001 *	−2.61	−0.82
0.9% Saline	Distilled Water	0.16	0.37	0.908	−0.74	1.05
	Saline-Water Mixture	−1.56	0.37	<0.001 *	−2.46	−0.67
Saline-Water Mixture	Distilled Water	1.72	0.37	<0.001 *	0.82	2.61
	0.9% Saline	1.56	0.37	<0.001 *	0.67	2.46
**(I_B_) Carbonisation**	**(J_B_) Carbonisation**	**Mean Difference (I_B_ − J_B_)**	**Std. Error**	**Sig.**	**95% Confidence Interval**	
					**Lower Bound**	**Upper Bound**
Distilled Water	0.9% Saline	−0.73	0.135	<0.001 *	−1.06	−0.41
	Saline-Water Mixture	−0.13	0.135	0.588	−0.46	0.19
0.9% Saline	Distilled Water	0.73	0.135	<0.001 *	0.41	1.06
	Saline-Water Mixture	0.60	0.135	<0.001 *	0.27	0.93
Saline-Water Mixture	Distilled Water	0.13	0.135	0.588	−0.19	0.46
	0.9% Saline	−0.60	0.135	<0.001 *	−0.93	−0.27

Sig.: Statistical significance. Significant differences on a level of *p* < 0.001 are marked with an asterisk (*).

## Data Availability

A full data set is available upon request to the corresponding author.
